# Comparison of regression imputation methods of baseline covariates that predict survival outcomes

**DOI:** 10.1017/cts.2020.533

**Published:** 2020-09-04

**Authors:** Nicole Solomon, Yuliya Lokhnygina, Susan Halabi

**Affiliations:** 1 Department of Biostatistics and Bioinformatics, Duke University, Durham, USA; 2 Duke Clinical Research Institute, Durham, USA; 3 Duke Cancer Institute, Duke University Medical Center, Durham, USA

**Keywords:** Missing data, regression imputation, proportional hazards model

## Abstract

**Introduction:**

Missing data are inevitable in medical research and appropriate handling of missing data is critical for statistical estimation and making inferences. Imputation is often employed in order to maximize the amount of data available for statistical analysis and is preferred over the typically biased output of complete case analysis. This article examines several types of regression imputation of missing covariates in the prediction of time-to-event outcomes subject to right censoring.

**Methods:**

We evaluated the performance of five regression methods in the imputation of missing covariates for the proportional hazards model via summary statistics, including proportional bias and proportional mean squared error. The primary objective was to determine which among the parametric generalized linear models (GLMs) and least absolute shrinkage and selection operator (LASSO), and nonparametric multivariate adaptive regression splines (MARS), support vector machine (SVM), and random forest (RF), provides the “best” imputation model for baseline missing covariates in predicting a survival outcome.

**Results:**

LASSO on an average observed the smallest bias, mean square error, mean square prediction error, and median absolute deviation (MAD) of the final analysis model’s parameters among all five methods considered. SVM performed the second best while GLM and MARS exhibited the lowest relative performances.

**Conclusion:**

LASSO and SVM outperform GLM, MARS, and RF in the context of regression imputation for prediction of a time-to-event outcome.

## Introduction

### Background

Clinical studies are often plagued by missing data. Unique patterns of missing data can occur due to dropout, singular missed follow-up visits, or missing singular data items during a study visit [1,2]. Rubin [3] established a classification of three types of incomplete data: missing completely at random when nonresponse is purely random; missing at random (MAR) when nonresponse is related to measured covariates; and non-ignorable missing where missing data are incomplete due to some association with unmeasured confounders [4–8].

There are various methods for handling missing data [9]. The simplest technique to handle missing data is the “complete case” analysis where only those observations with no missing records are included in the final analysis, which is the default in several packages. The complete case deletion approach may produce biased results, unless the missingness mechanism is missing completely at random [10]. In addition, it has the serious drawback of dropping a significant proportion of the original sample size and compromising the statistical power of the study.

Maximal data usage can lead to a more precise point and interval estimates with less bias, exclusion of fewer covariates and observations, and more representative analyses if the correct mechanism of missingness is assumed [4,5,11]. Simple imputation methods have been suggested (that is, replacing the missing value with the mean, median, mode, or last value carried forward), but these have the caveat of reducing standard error of the covariates. There are several approaches for handling missing data that offer better estimates and measures of uncertainty. These include regression [12], maximum likelihood methods, including the expectation–maximization (EM) algorithm, Bayesian methods [13], matching approaches (such as augmented inverse probability weighting [14], and multiple imputations [15,16]). Several authors provide general guidelines on how to handle missing data in clinical trials [17,18] and methods that impute longitudinal covariates and outcomes [19]. The reader is referred to these references for additional details [4,5,11,20,21].

### Motivating Example

We were interested in developing a prognostic model of overall survival (OS) based on baseline covariates of patients enrolled on the pivotal Phase III TROPIC trial that led to the Food and Drug Administration and European Medicines Agency approval of cabazitaxel for treating men with advanced prostate cancer [22,23]. Seven-hundred and fifty-five patients on the TROPIC trial were randomized with equal probability to either cabazitaxel or mitoxantrone groups. The study was designed to detect a hazard ratio of 0.75 in the cabazitaxel compared to the mitoxantrone groups, assuming 0.90 power, a two-sided significance level of 0·05, and a median OS of 8 months in the mitoxantrone group. Patients were followed until the target of 511 deaths had occurred. The primary purposes of the model are to predict the probability of OS at different time points and to identify prognostic risk groups in men who failed first-line chemotherapy. The clinical trial had a 67% event rate (32% of patients were censored) on the primary outcome (OS). However, several established prognostic factors of OS were incomplete with missing proportions exceeding 15% in 6 of the 23 covariates that were considered for development of the prognostic model. This presented a substantial challenge as a complete case approach would have resulted in an almost 30% loss in the number of patients included in constructing the prognostic model of OS.

Regression imputation has not been extensively studied and the lack of certainty in the most efficient imputation technique served as motivation for this simulation study. In particular, the choice of regression model or algorithm to impute missing values of incomplete covariates warrants further investigation. Regression imputation is a procedure to predict an incomplete covariate’s missing values based jointly on the outcome of interest and the complete explanatory covariates. Generalized linear model (GLMs) are extensions of the classical linear model to non-normal response variables and can be used to model broad types of response distributions [24,25], handle both categorical and continuous variables, provide a straightforward model interpretation of regression coefficients, and incorporate commonly known variable selection procedures [26]. The least absolute shrinkage and selection operator (LASSO) is a linear regression model that uses *l*_*1*_ penalty for subset selection [27], and shrinking parameters ***β*** = (*β*_*1*_, *β*_*p*_) for overcoming the problem of overfitting [28–30]. The multivariate adaptive regression splines (MARS) is a flexible regression procedure for the modeling of high-dimensional data [31–33]. The nonparametric support vector machine (SVM) is a learning method that has been applied for classification of binary outcomes or regression of continuous outcomes or to make predictions and has been shown to be effective in some pattern recognition and other applications [34–39]. Lastly, random forests (RFs) are a learning method for classification, among other applications. The classification is conducted by generating numerous decision trees, which are randomly sampled from the training data, and identifying the most popular class for a given input [40]. Several RF approaches exist to impute missing data and their strengths include handling mixed data types, nonlinearity, and high dimensions [41].

We chose these five methods because they are popular and have been used extensively in machine learning and the medical literature. We compare these five specific regression approaches or algorithms in imputing missing baseline covariates. The remainder of this article is organized in the following manner. We describe the simulation of data and imputation of missingness in the methods section. We then apply the regression imputation methods to our motivating example and, lastly, we conclude the paper with summarizing remarks and recommendations.

## Methods

Complete datasets were generated, a missingness mechanism was applied, and the resulting incomplete observations were imputed under each regression method. The study was conducted under the assumptions of non-informative censoring, MAR data, and an outcome variable that adheres to the proportional hazards model. All simulations and analyses were performed in R version 3.4.4.

### Complete Datasets

The true failure and censoring times *T*_*i*_ and *C*_*i*_ were drawn from the Weibull distribution [42,43]. It is not uncommon in cancer trials to observe OS times fit a Weibull distribution. In fact, investigation of survival curves following an exponential distribution or a Weibull distribution confirmed that the TROPIC data can be matched to a Weibull distribution with appropriate parameters; draws from a Weibull distribution with appropriate parameters closely followed the observed the TROPIC survival curves. Thus, we used the Weibull distribution since it matched the distributions observed in the motivating TROPIC data. The censoring distribution was independent and non-informative. The observed time and censoring indicators were defined as *Y*_*i*_ = min(*T*_*i*_ *, C*_*i*_) and *δ*_*i*_ = *I*(*T*_*i*_ ≤ *C*_*i*_). These times achieve pre-specified median survival times on each equally proportioned strata of the primary predictor, treatment arm (*ARM*). Specifically, the experimental arm and control were simulated to have a median survival time of 15 months and 11 months, respectively.

The covariates from the TROPIC trial (training set) were chosen to serve as the basis for the distributions of the simulated explanatory variables (Table 1). These predictors were chosen primarily because they are established prognostic factors of OS [44,45] in prostate cancer patients and because of their proportion of missingness. They included categorical variables’ Eastern Cooperative Oncology Group performance status (*ECOG* ∈ {0, 1, 2}), an indicator of whether progression occurred < 6 months since the last Taxotere session (*PROG* ∈ {0, 1}), pain at baseline (*PAIN* ∈ {0, 1}), an indicator of White race (*WHITE* ∈ {0, 1}), indicator of chemotherapy treatment (*CHEMO* ∈ {0, 1}), indicator of measurable disease (*MEAS_DIS* ∈ {0, 1}). Continuous variables included age (*AGE*), hemoglobin concentration (*HGB*), the log of the concentration of alkaline phosphatase (*LALP*), log of the concentration of prostate-specific antigen (*LPSA*), time on hormone (*TIME_HORMONE*), years since diagnosis (*YRSINCEDIAG*), and body mass index (*BMI*).

**Table 1.** Distribution of chosen baseline covariates from the TROPIC trial

Discrete variables	% No (0)	% Yes (1)	% (2)*	% Missing
ARM	0.5	0.5		0.0
ECOG	0.34	0.58	0.08	0.0
PROG	0.11	0.89		0.0
CHEMO	0.68	0.32		0.0
PAIN	0.49	0.51		16.2
WHITE	0.16	0.84		0.0
MEAS_DIS	0.47	0.53		0.0

*Continuous Variables*	*Mean*	*Stdev*	*Range*	
AGE	67.3	7.9	47.0–92.0	0.0
HGB	120.9	14.0	76.4–185.0	1.6
LALP	5.07	0.91	0.67–8.94	2.6
LPSA	4.86	1.59	0.47–8.97	1.8
BMI	27.85	4.65	17.48–46.79	0.20
TIME_HORMONE	4.32	2.81	0.20–16.30	3.4
YRSINCEDIAG	5.8	4.0	0.1–23.6	7.7
*Defined only for ECOG category = 2				

### Simulation of Data

All simulated covariates were drawn from a multivariate normal (MVN) distribution in order to induce correlation among them (the covariance matrix can be found in the Supplementary Materials). Inverse transform sampling was applied to those MVN draws corresponding to categorical variables in order to convert them from continuous values to binary or categorical factors. The specific event probabilities for each categorical variable’s levels are listed in the Supplementary Materials, as are the means and variances of the continuous covariates.

Simulations were conducted under combinations of each of the following characteristics: sample size (*N =* 200, 500, or 1000), percent censoring (*C =* 10% or 30%), and percent missing data (*M =* 5%, 10%, or 15%). These aspects result in a total of 6 complete datasets (combinations of *N* and *C*), 18 incomplete datasets (combinations of *N*, *C*, and *M*), and, after applying the 5 imputation regression methods, 90 imputed datasets (Table 2).

**Table 2.** Parameter settings for simulation studies

Parameter	Levels		
N	200	500	1000
C	10%	30%	
M	5%	10%	15%

N, sample size; C, censoring percentage; M, missing percentage.

The sample sizes and censoring proportions were chosen foremost for their realistic values in oncology trials. A sample of 500 patients is not an uncommon size of a (training) dataset; in fact, the basis of this simulation study is the 507 observations from a training subset of the TROPIC trial. The smallest size of 200 patients was included to demonstrate each method’s performance in a smaller sample size context. The larger censoring proportion was chosen to approximate TROPIC while the smaller value was chosen to allow for performance comparisons under “ideal” circumstances. The *N* and *C* levels considerably differ in size to make obvious any differences in performance across these dataset characteristics. The levels of *M* were chosen to represent favorable (5%) [46], average (10%), and undesirable (15%) rates of missing data [47].

### Incomplete Datasets

Three copies of each complete dataset were created upon which each of the three pre-specified levels of missingness were applied. This produced 18 incomplete datasets from the combinations of sample size, percent censoring, and percent missing. Of the 13 explanatory variables at baseline, three were chosen to be missing at random: those variables that represent progression shortly after Taxotere administration (*PROG*), hemoglobin (*HGB*), and alkaline phosphatase (*LALP*). Missing values were imposed by constructing logistic models to regress the probability of missingness on all of the complete variables. These probabilities were then used as the event rate of random Bernoulli draws to indicate missing or not missing, thereby creating a MAR pattern. This process was repeated 5000 times for each of the 18 characteristic combinations to obtain sampling distributions of the statistics of interest. More information is available in the Supplementary Materials Sect. 2 on the missingness mechanism.

### Choice of Imputation Model

The key to a successful regression analysis is the assumption that the model used to link the outcome to the explanatory covariates fits the data well. White and Royston conducted simulation studies to determine the most efficient form by which survival should be incorporated into a regression imputation model [12]. They assessed several representations of survival time *T* including linear *T*, polynomial *T*^2^, log(*T*), the cumulative baseline hazard 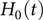, among others [12]. Moreover, they endorsed regression of the incomplete covariate *X*_*j*_ *, j =* 1,2,3, on the complete covariates *Z*, the remaining 10 TROPIC covariates previously mentioned and *ARM*, and on survival represented by the event indicator *δ* and the Nelson–Aalen estimate of the baseline cumulative hazard function 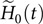. This model performed at least as well as the other regression forms studied, often with the lowest bias and highest power [12].

Following the suggestion of White and Royston [12], we consider the following model where the incomplete covariate *X*_*j*_ is regressed on the complete covariates *Z* and on survival represented by both the event indicator δ and the estimated baseline cumulative hazard function 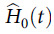:(1)
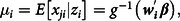
where 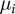 is the mean of the response for patient *i*, *g* is identity when *X*_*j*_ is continuous or is the inverse logit transformation when *X*_*j*_ is binary, ***w***_***i***_ = [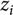, 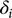, 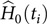], and ***β*** is the vector of unknown regression coefficients. This general form of regression imputation model is adopted for our simulation study herein, and employs the Kalbfleisch and Prentice estimate 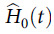. The Nelson–Aalen estimate [48,49] 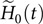 is ideal in an active clinical trial but the lack of ties in the simulated data supports the use of the Kalbfleisch and Prentice estimate [50].

### Imputation

For each of the 18 sets of incomplete data, imputation models were fitted based on Eq. 1. This model was incorporated into each of the five regression algorithms of interest; i.e., the formula used for each regression method’s imputation was Eq. 1. First, the baseline cumulative hazard 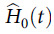 was iteratively calculated for each dataset conditioning on complete covariates, as described in White and Royston [12]. Logistic regression models were then fitted for the incomplete binary covariate and linear regression models for the incomplete continuous covariates. Specifically, the GLM and LASSO models utilized a logit link for the binary incomplete covariate and the identity link for the continuous incomplete covariates. The difference between these ultimately comes down to penalization and shrinking of model coefficients by LASSO, thereby employing a potentially smaller model for imputation. The tuning parameter for LASSO was fit by 10-fold cross-validation and selection of the parameter which was within one standard error of the parameter that minimizes the mean cross-validated error. The tuning of SVM was achieved using tune.svm to conduct 10-fold cross-validation and identify the best regularization parameter and best kernel coefficient via a grid search across a range of sampling space; in this case, “best” refers to those parameters that yielded the most accurate model. We chose to use this “one-standard-error” rule in order to select a more parsimonious imputation model that still controls cross-validation error [27]. The RF method was tuned to identify the optimal number of variables to randomly choose as model candidates at each split, which minimizes the out-of-bag estimated error rate.

For each model, the 11 complete covariates, represented by *Z*, the event indicator *δ*, and 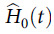 acted as predictors of the missing variable *X*_*j*_ to be imputed:
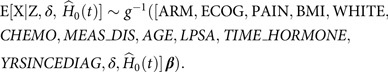



Function *g* is as defined in Sect. 2.2. The GLM, LASSO, MARS, SVM, and RF models were fitted in R via the functions glm, cv.glmnet, earth, svm, and impute.rfsrc, respectively [51–55]. All the programs were written by the first author in R version 3.4.4 and are available at: https://duke.box.com/s/5bn0f5gk5xmh3ova92rg9nzn9s1rsnil.

These fitted models then provided the predicted values for the missing observations of each incomplete covariate. To avoid underestimation of the variability in the prediction procedure, a residual error term was drawn from a normal distribution with mean 0 and standard deviation equal to that of the incomplete covariate’s observed values, *e ~ N*(0*, sd*(*X*)), and added to the predicted imputation value. In this manner, a total of 90 imputed datasets were obtained, each containing 5000 simulations.

### Assessment of Imputed Data

Each of the five regression method’s imputed datasets was utilized to fit the Cox proportional hazards model predicting the time to death or censoring using six covariates: the three imputed covariates *X = (PROG, HGB, LALP)* and three complete covariates 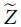 = *(ARM, ECOG, AGE)*:
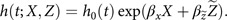



The methods were then compared by evaluating the six estimated regression coefficients 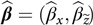. Generalized summary statistics were chosen to assess each method’s global performance, specifically any error in the estimation of 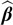. To represent each method’s efficiency across the 6 (*V*) covariates and 5000 (*S*) simulation runs, the average absolute proportional bias, the average proportional mean square error (MSE), the average mean square prediction error (MSPE), the average median absolute deviation (MAD), and the average minimum 95% probability coverage (mPCOV) were computed in the following manner:

1. Average absolute proportional bias:
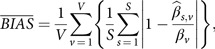

2. Average proportional MSE:
  First, define MSE per Rubin’s rules for multiple imputations [56]:

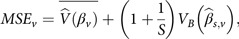

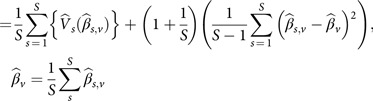


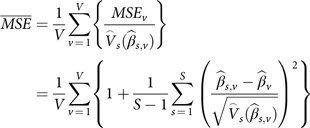

  or 
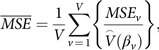

3. Average mean squared prediction error (MSPE):
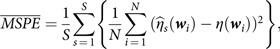
where 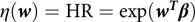,
4. Average MAD:
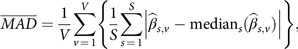

5. Average minimum 95% probability coverage (mPCOV):
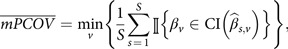

where the confidence interval is a standard 95% confidence interval.

The results of each of the 5 regression methods for the 18 combinations of sample size, percent censoring, and percent missing were averaged across the 5000 simulated datasets for each performance statistic. Furthermore, to heuristically compare the methods’ performances, each regression method was assigned a rank within each level of missing percentage, for each statistic. Hence, a total of 75 ranks were assigned for each combination of *N* and *C*, across the 3 levels of missingness, the 5 summary statistics, and the 5 imputation methods. The mode of these ranks was then taken across missing percentage for a total of 25 final ranks per combination of *N* and *C.* That is, each of the 5 regression methods was given a single rank for each of the 5 statistics within the 6 combinations of sample size and censoring proportion. Thus, a total of 30 final rankings were assigned to each method. The number of times a method ranked 1^st^ or 2^nd^ was considered “good” performance.

## Results

### Simulation Results

The relative ranking of each regression method for each summary statistic and each combination of *N* and *C* is presented in Table 3. The results therein are based on summary statistics averaged across the 5000 simulations executed for each simulation scenario. When *N* = 200 and *C* = 30%, with the least number of events (140), SVM had the lowest bias followed by LASSO, MARS, RF, and GLM. LASSO ranked number 1 in terms of every summary statistic with the exception of bias and mPCOV (Table 3). SVM and LASSO most frequently achieved “good” ranks of 1 or 2 with frequencies 24/30 and 23/30, respectively (Table 3).

**Table 3.** Relative rank of regression imputation methods by simulation scenario (number of events)

Dataset	Statistic	GLM	LASSO	MARS	SVM	RF
140 events *N* = 200 *C* = 30%	Bias	5	2	3	1	4
	MSE	4	1	5	2	3
	MSPE	4	1	3	2	5
	MAD	4	1	3	2	5
	mPCOV	3	5	4	1	2
180 events *N* = 200 *C* = 10%	Bias	4	1	5	2	3
	MSE	4	1	5	3	2
	MSPE	3	1	4	2	5
	MAD	4	1	3	2	5
	mPCOV	3	2	4	1	5
350 events *N* = 500 *C* = 30%	Bias	4	1	5	3	2
	MSE	4	1	5	2	3
	MSPE	4	1	3	2	5
	MAD	4	1	3	2	5
	mPCOV	1	5	4	3	2
450 events *N* = 500 *C* = 10%	Bias	5	1	3	2	4
	MSE	4	3	5	1	2
	MSPE	3	1	4	2	5
	MAD	5	1	3	2	4
	mPCOV	3	5	4	2	1
700 events *N* = 1000 *C* = 30%	Bias	3	1	5	4	2
	MSE	4	2	5	1	3
	MSPE	4	1	3	2	5
	MAD	4	1	3	2	5
	mPCOV	1	5	4	3	2
900 events *N* = 1000 *C* = 10%	Bias	4	1	5	3	2
	MSE	4	1	5	2	3
	MSPE	4	1	3	2	5
	MAD	4	1	3	2	5
	mPCOV	2	5	4	3	1
(Freq. in Top 2)/30		3	24	0	23	10
(Freq. in Bottom 2)/30		21	5	18	1	15
(Freq. in Bottom 3)/30		27	6	12	7	20

MSE, mean squared error; MSPE, mean squared prediction error; MAD, median absolute deviation; mPCOV, minimum 95% probability coverage.

It is worth noting that all methods performed poorly with respect to bias, regardless of percent missing, as all methods overestimated the model parameters considerably (Table 4). Increasing percent of missingness caused a decrease in performance in GLM and MARS in some statistics. LASSO was robust to increasing the proportion of missingness at both levels of censoring (*C* = 30, 10%). GLM and RF remained constant in relative performance of MAD. SVM improved in MSPE with increasing percent missingness (Table 4).

**Table 4.** Summary statistics of simulations by regression imputation method and percent missing

	5%					10%					15%				
% Missing	GLM	LASSO	MARS	SVM	RF	GLM	LASSO	MARS	SVM	RF	GLM	LASSO	MARS	SVM	RF
(a) 140 events (*N* = 200 *C* = 30%)															
P.Bias	2.125	2.056	1.963	1.896	2.061	2.274	2.093	2.357	2.063	2.191	2.471	2.099	2.159	2.051	2.230
P.MSE	2.171	2.148	2.191	2.144	2.150	2.244	2.122	2.297	2.178	2.184	2.283	2.131	2.453	2.210	2.216
MSPE	2.122	2.051	2.113	2.083	2.159	2.104	1.963	2.046	2.029	2.173	2.073	1.914	2.019	1.981	2.187
MAD	0.470	0.442	0.460	0.458	0.472	0.470	0.424	0.447	0.445	0.477	0.473	0.409	0.437	0.431	0.479
mPCOV	0.768	0.772	0.765	0.764	0.769	0.763	0.752	0.762	0.771	0.768	0.758	0.721	0.743	0.769	0.767
(b) 180 events (*N* = 200 *C* = 10%)															
P.Bias	1.819	1.670	1.699	1.804	1.741	1.738	1.672	1.779	1.734	1.771	1.850	1.540	2.074	1.739	1.789
P.MSE	2.188	2.144	2.209	2.147	2.150	2.203	2.143	2.334	2.156	2.154	2.239	2.133	2.379	2.186	2.180
MSPE	2.008	1.983	2.039	1.995	2.081	1.974	1.893	1.980	1.925	2.087	1.930	1.808	1.950	1.882	2.094
MAD	0.480	0.456	0.473	0.469	0.486	0.473	0.435	0.455	0.452	0.484	0.472	0.419	0.448	0.439	0.482
mPCOV	0.761	0.763	0.756	0.763	0.758	0.761	0.764	0.761	0.768	0.760	0.760	0.729	0.759	0.760	0.761
(c) 350 events (*N* = 500 *C* = 30%)															
P.Bias	1.175	1.096	1.116	1.122	1.071	1.124	1.038	1.389	1.109	1.081	1.185	1.063	1.264	1.079	1.068
P.MSE	2.127	2.143	2.180	2.124	2.120	2.172	2.108	2.527	2.143	2.141	2.194	2.136	2.770	2.157	2.192
MSPE	1.783	1.758	1.787	1.775	1.834	1.751	1.688	1.735	1.726	1.849	1.720	1.632	1.701	1.666	1.868
MAD	0.454	0.434	0.444	0.439	0.455	0.448	0.410	0.429	0.424	0.455	0.446	0.392	0.418	0.410	0.456
mPCOV	0.784	0.783	0.780	0.789	0.785	0.782	0.734	0.752	0.778	0.788	0.789	0.654	0.722	0.745	0.776
(d) 450 events (*N* = 500 *C* = 10%)															
P.Bias	7.268	5.901	6.515	5.946	6.653	4.722	5.013	2.435	2.494	2.678	7.694	3.671	4.311	4.976	4.140
P.MSE	2.189	2.152	2.248	2.146	2.149	2.190	2.155	2.599	2.181	2.179	2.229	2.172	3.052	2.168	2.190
MSPE	1.719	1.694	1.728	1.711	1.770	1.672	1.630	1.680	1.661	1.782	1.642	1.574	1.628	1.604	1.794
MAD	0.459	0.437	0.450	0.444	0.459	0.452	0.414	0.438	0.432	0.460	0.447	0.398	0.424	0.416	0.459
mPCOV	0.754	0.767	0.759	0.759	0.756	0.757	0.733	0.742	0.758	0.761	0.748	0.662	0.684	0.734	0.757
(e) 700 events (*N* = 1000 *C* = 30%)															
P.Bias	0.964	0.941	0.983	0.872	0.948	0.948	0.908	1.166	0.987	0.939	1.003	0.947	1.313	1.033	0.955
P.MSE	2.082	2.070	2.131	2.081	2.065	2.131	2.095	3.012	2.092	2.115	2.178	2.100	3.722	2.120	2.156
MSPE	1.701	1.680	1.705	1.694	1.754	1.671	1.628	1.647	1.641	1.769	1.646	1.569	1.614	1.603	1.787
MAD	0.451	0.428	0.440	0.436	0.453	0.446	0.406	0.427	0.422	0.454	0.445	0.390	0.417	0.408	0.454
mPCOV	0.786	0.780	0.786	0.783	0.782	0.782	0.664	0.735	0.758	0.781	0.755	0.560	0.712	0.690	0.744
(f) 900 events (*N* = 1000 *C* = 10%)															
P.Bias	0.915	0.975	1.027	0.987	0.979	1.118	0.843	1.365	1.010	0.942	1.138	0.821	1.100	1.067	0.849
P.MSE	2.221	2.169	2.268	2.188	2.173	2.208	2.160	3.570	2.184	2.221	2.272	2.176	4.770	2.208	2.250
MSPE	1.646	1.629	1.659	1.640	1.709	1.606	1.573	1.601	1.588	1.725	1.569	1.521	1.566	1.533	1.741
MAD	0.443	0.425	0.438	0.433	0.448	0.439	0.404	0.423	0.417	0.450	0.436	0.386	0.411	0.403	0.448
mPCOV	0.750	0.755	0.753	0.754	0.754	0.753	0.665	0.723	0.744	0.755	0.726	0.507	0.690	0.665	0.730

When *N* = 500, LASSO ranked first in all summary statistics across both levels of censoring (*C* = 10%, 30%) except for mPCOV, where it continued its poor relative performance seen at *N* = 200, and for MSE when *C* = 10%. As missingness improved at *C* = 30%, all methods excluding MARS improved in bias and all methods maintained their relative rank in MSE and MAD. At *C* = 10% the relative ranking for LASSO, MARS, and SVM remained constant or improved in all summary statistics as the level of missingness increased. GLM either worsened or remained constant in all summary statistics. Overall, SVM outperformed MARS in all performance statistics. MARS demonstrated the worst absolute proportional bias and MSE.

Lastly, LASSO came in the top rank for the relative performance of bias, MSPE, and MAD in the *N* = 1000 datasets. SVM and LASSO were the best approaches at both *C* = 30% and 10% but MARS overtook SVM in bias with the 900 events at *C* = 10%. In fact, SVM ranked second for all statistics at *N* = 1000 except bias at *C* = 10%. GLM took top rank in mPCOV. As observed in the smaller datasets, GLM’s performance was otherwise consistently poor across summary statistics and censoring proportions. All methods maintained constant relative rank in MSPE and MAD at *C* = 30% and in MSE, MSPE, and MAD at *C* = 10%. GLM failed to improve in rank in any statistic at *C* = 10% while MARS did not fall in rank in any statistic.

In general, LASSO never fell below the first ranking in relative performance of bias, MSPE, and MAD for most combinations of sample size and censoring proportion. However, it consistently was the worst in relative performance of minimum probability coverage (Table 3). LASSO is the most sensitive to rising missing percentages in terms of largest reduction in mPCOV; at *N* = 500, *C* = 30% it falls from 0.792 coverage at *M* = 5% to 0.633 at *M* = 15% compared to GLM which falls only from 0.786 to 0.731 (Table 4). Additionally, SVM never fell below second rank in MSE, MSPE, and MAD. MARS consistently performed poorly. GLM never rose above fourth rank in relative performance of MSPE and MAD, and always demonstrated the highest mPCOV rank.

It should be noted that the age covariate was exceptionally biased by all methods on average under all simulation parameters. When age is excluded from the calculation of average proportional absolute bias, the average bias for all methods at *N* = 1000 was closer to 0.20, at *N* = 500 it was approximately 0.30, and at *N* = 200, it averaged at 0.9 for *C* = 10% and 1.0 for *C* = 30%.

Overall trends in performance of each method were observed with decreasing censoring (Table 4). When contrasting the scenarios with lower event rates (*C* = 30%) versus higher event rates (*C* = 10%), it can be seen that all methods observed a drop in bias and otherwise approximately constant performance in MSE, MSPE, MAD, and mPCOV. The one exception is MARS which observed increases in MSE at *C* = 10% versus 30%, primarily when *M* = 15%. LASSO’s mPCOV was higher at *C* = 30% compared to *C* = 10%.

**Fig. 1.**
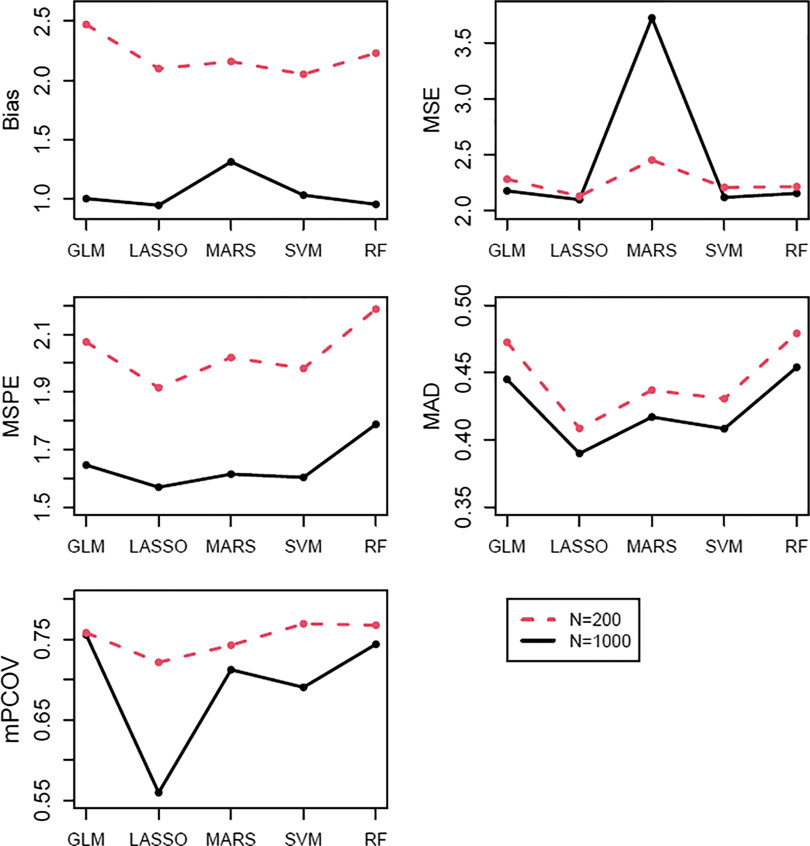
Performance of regression imputation methods for each summary statistic in simulations where *C* = 30% and *M* = 15%.

**Fig. 2.**
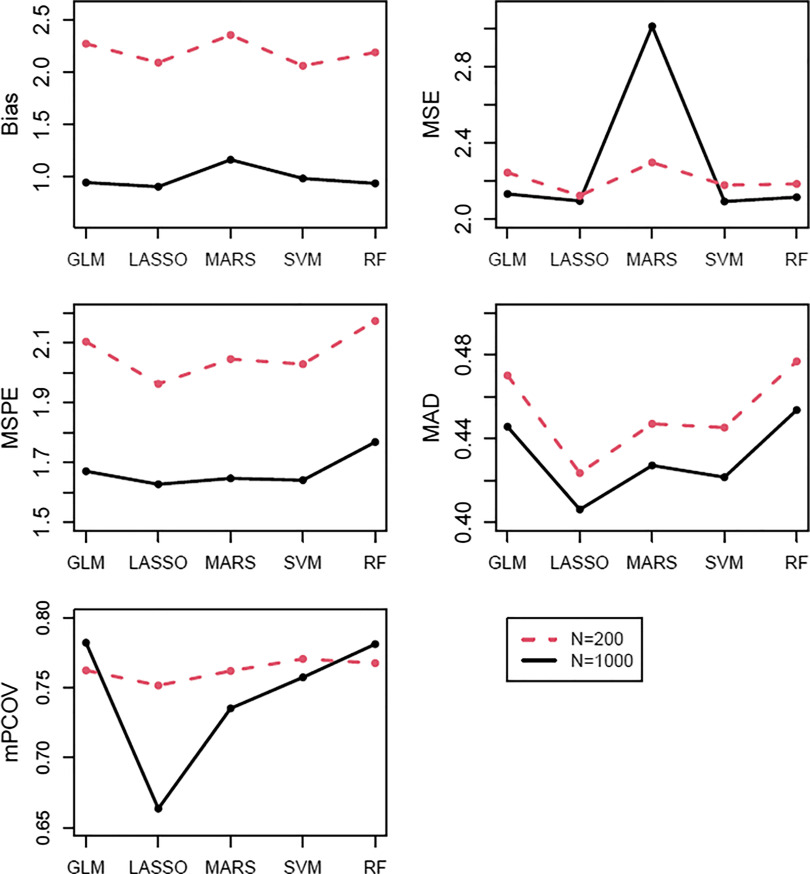
Performance of regression imputation methods for each summary statistic in simulations where *C* = 30% and *M* = 10%.

The trend in relative performance with an increasing sample size can be seen at *C* = 30%, *M* = 15%, and *C* = 30%, *M* = 10% in Figs. 1 and 2, respectively. The trend was of decreases in bias, MSPE, MAD, and mPCOV, and small increases in MSE for all methods. MARS demonstrated a noticeable rise in MSE with increasing *N*. The trend of decreasing mPCOV with increasing sample size is due to the narrowing of the confidence intervals.

Lastly, holding both sample size and censoring percentage fixed resulted in similar performance by all methods as missingness increases: approximately constant performance in MAD and MSE – excluding SVM which saw higher MSEs at higher missing rates – and drops in MSPE and mPCOV (Table 4). For the bias statistic, SVM and LASSO typically were constant or improved, GLM improved for all but the *C* = 10% and *N* = 500, 1000 scenarios, and MARS often worsened in relative performance. Overall, all methods became less sensitive to missingness, especially in MSE, MSPE, and MAD, as the sample size increased.

Trends in behavior of each statistic were also noted. As expected, bias improved and decreased with increasing sample size for all five methods, while MSE remained constant; i.e., variability increased. MAD was fairly robust to all three simulation parameters; for any value of any parameter, it was near 0.47 for any of the five imputation methods. mPCOV was approximately constant for any sample size or censoring proportion. As expected, the remaining statistics improved with increasing *N*. Within a fixed sample size MSPE and MAD were robust to changing censoring proportions while bias and MSE improved with higher event counts; i.e., with lower *C*.

### Motivating Example

Our first step was to assess the mechanism of missingness so an appropriate imputation method can be applied. This is one of the most challenging tasks as data that aid in determining the type of missingness are often not collected in studies. This is not a unique problem to the TROPIC trial, as such information is often overlooked in clinical trials and studies. The underlying missing covariate data mechanism was not missing completely at random, but rather was missing at random. This was a reasonable assumption as missing completely at random is highly unlikely to hold in all but the simplest experimental settings since missingness in one variable is frequently related to whether another variable is also missing. MAR can be empirically confirmed by assessing the correlation between the missingness in a given covariate and the observed values of another covariate. Thus, we assumed the covariate values are MAR because the correlation between missing values of given covariates and the observed values of other covariates was substantial. For example, missing values of baseline hemoglobin and observed values of measurable disease had a correlation of approximately 40%. Non-ignorable missingness was deemed unlikely due to the capture of an extensive set of covariates which considerably improve the efficiency of imputation procedures.

We imputed the missing data based on 12 baseline variables in a manner identical to the methods described above; the baseline cumulative hazard was estimated and then included with the set of complete covariates in a regression model to predict and impute the missing values of the incomplete variables. This was done once for each of the five regression techniques. The incomplete variables in the TROPIC training set were: *PAIN*, *HGB*, *LALP*, *LPSA*, *BMI*, *TIME_HORMONE*, and *YRSINCEDIAG*. The complete variables used to impute the missing values were: *ECOG*, *WHITE (for race)*, *MEAS_DIS*, *AGE*, *CHEMO*, *VISCMET*, δ, and 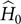 where *VISCMET* is an indicator for whether the patient had metastases in so-called “visceral” locations – not in the lung, liver, bone, lymph node, etc. When we fit a model with the complete records, the time-dependent area under the curve is 0.77 (95% CI = 0.51, 0.78). When we fit a model with the imputed data, the time-dependent areas under the curve are 0.75 (95% CI = 0.51, 0.77 95% CI), 0.76 (95% CI = 0.51, 0.77), 0.77 (95% CI = 0.51, 0.77), 0.77 (95% CI = 0.51, 0.77), and 0.77 (95% CI = 0.51, 0.77) for the SVM, LASSO, RF, GLM, and MARS approaches, respectively.

Since the results of these simulations were unavailable at the time of model building for the TROPIC dataset, SVM was chosen for imputing the missing variables in the prognostic model of OS due to its superiority in some application of pattern recognition and machine learning methods [22].

## Discussion

In this article, we investigated the performance of five different regression methods, GLM, LASSO, MARS, and SVM, when applied for the purpose of single imputation of missing baseline covariates in predicting survival outcomes and estimating covariate effects. Linear and logistic models were constructed under these modeling methods for the purpose of imputing missing values of normal and binary explanatory variables respectively. This process was completed in the context of time-to-event outcomes that are subject to right censoring.

We focused on the survival outcome as this was our motivation example and we adopted the White and Royston approach [12]. The simulations conducted here were based on the assumption of missing at random. Through our simulation studies, we have demonstrated the feasibility of applying regression imputation approaches for missing baseline covariates that predict survival outcomes that are subject to right censoring. The results of the simulation show that SVM and LASSO outperformed GLM and MARS. This can be attributed to LASSO’s ability to both efficiently estimate regression parameters as well as shrink the parameters. This provided more accurate predictions of missing observations than the other methods and so led to more accurate results in the fitted Cox proportional hazards model.

On an average, LASSO and SVM were robust regardless of sample size, censoring percentage, and missing percentage. We note also that none of the regression or classification methods appeared to differ in summary statistic performance between continuous and categorical covariates. They produced the most efficient statistic value for the majority of performance settings. The one exception was mPCOV which could be due to LASSO’s shrinkage; relative to the other methods it applies a greater magnitude of shrinkage to some covariates and so has tighter confidence intervals. Across the six combinations of sample size and censoring, MARS had the least impressive performance relative to the other regression methods. Though differences in performance were observed, the differences were not large in magnitude. It is possible that an adjustment to the signal-to-noise ratio may improve the apparent performance of the flexible nonparametric models MARS and RF. Additional research could investigate this further.

All simulations were run in parallel and so GLM, LASSO, and MARS took very little time to complete the 5000 simulations for each scenario; less than 4 min for even the largest sample size of 1000. Computationally, as expected, GLM took the least time to complete the simulations – less than 15 min for all 18 scenarios, with MARS requiring little additional time comparatively – less than 20 min. Each of these two methods took less than a minute to run all of the simulations for any given set of simulation parameters. LASSO took more time than MARS or GLM combined but was still very quick overall; each scenario required at most 4 min to complete all 5000 simulations. SVM was considerably slowed down by the tuning step and even when run in parallel across a multi-core processor required significantly more time than the other three methods combined; each scenario took more than 1 h. RF took less time to run than SVM, but more than the other three methods; anywhere from 8 to 30 min per scenario.

The study presented herein has several limitations. First, simulations were conducted only on a low-dimensional dataset that included 14 covariates. Performance of the five regression methods may have varied more significantly in a high-dimensional dataset. Second, only three incomplete variables were imputed. Third, we have not compared the regression approaches with multiple imputations nor maximum likelihood methods. Nonetheless, this simulation study is important as it has shown that SVM and LASSO are the preferred options for regression imputation in the context of baseline covariate that is missing in predicting survival outcome under Eq. 1 and the simulation assumptions. LASSO is recommended for both its higher predictive accuracy and computational efficiency. Future research should study: the effects of stronger and weaker correlations among the covariates, impact of mixed distributions of binary, categorical and continuous covariates, the performance in a high-dimensional dataset with numerous covariates, the performance under multiple imputation rather than single imputation, and longitudinal measures of the covariates and outcomes.

Missing data is inevitable in data in medical and other areas of research. Handling missing data is a complex and a challenging task. This is due both to the fact that the type of missingness mechanism is usually unknown and that the assumptions one makes about the missingness and the mechanism cannot be definitively checked from the observed data. It is highly recommended that data elements that help assess the type of mechanism be collected at the design stage so that data can be carefully assessed and assumptions checked before one embarks on an imputation approach. Investigators are encouraged to thoroughly check their data and assumptions, implement appropriate methods for imputing the missing data, and perform sensitivity analysis so valid inferences can be made.

## Supplementary material

For supplementary material accompanying this paper visit https://doi.org/10.1017/cts.2020.533. click here to view supplementary material
