# Supplementary material for: Comparison of regression imputation methods of baseline covariates that predict survival outcomes
Source: J Clin Transl Sci. 2020 Sep 4;5(1):e40. doi: 10.1017/cts.2020.533 (PMC8057424; doi:10.1017/cts.2020.533)
Supplement: Supplementary file 1 [file S2059866120005336sup001.docx]

Supplementary Materials for “Comparison of regression imputation methods of covariates that predict survival outcomes”

Nicole Solomon, Yuliya Lokhnygina and Susan Halabi

**Section 1.** *Simulated Data Generation*

All covariates were initially generated from a multivariate normal (MVN) distribution to induce correlations among all covariates. The covariance matrix listing both the variances and covariances is shown in Table S1 below. The MVN sampling draws corresponding to categorical covariates were then obtained by inverse transform sampling using their appropriate means and standard deviations.

The final simulated covariates were distributed as follows:

- Treatment arm was drawn from a Bernoulli (BERN) distribution with success probability 0.5: ARM ~ BERN(0.5)
- Eastern Cooperative Oncology Group performance status was drawn from a multinomial (MN) distribution with 3 possible categories: ECOG ~ MN{0, 1, 2} with probabilities {0.3, 0.6, 0.1}
- Age was drawn from a normal (N) distribution with mean 67 and variance 60:
  AGE ~ N(67, 60)
- Indicator of progression < 6 months since last taxotere session: PROG ~ BERN(0.9)
- Hemoglobin: HGB ~ N(120, 190)
- Log(alkaline phosphatase): LALP ~ N(5.05, 0.80)
- Pain at baseline: PAIN ~ BERN(0.52)
- Log(prostate specific antigen): LPSA ~ N(4.7, 2.56)
- White race: WHITE ~ BERN(0.8)
- Chemotherapy treatment: CHEMO ~ BERN(0.33)
- Measurable disease: MEAS_DIS ~ BERN(0.54)
- Time on hormones: TIME_HORMONE ~ N(4.2, 7.5)
- Years since diagnosis: YRSINCEDIAG ~ N(5.7, 15.5)
- BMI: BMI ~ N(27.8, 20.5)

The sampling draws of the last three covariates, TIME_HORMONE, YRSINCEDIAG and BMI, were all shifted up in magnitude to bring their range within realistic bounds.

The endpoint for the simulation study was a time-to-event endpoint subject to right censoring to represent overall survival. The event times were drawn from a Cox-Weibull distribution as defined in Bender et al (2005).^1^ The median event times for the control (*j=1*) and experimental (*j=2*) arms were set to $m_{1}=11$ and $m_{2}=15$ months respectively, similar to the clinical trial TROPIC.^2^ In order to both relate all covariates to the survival time and to achieve these median times, the squared difference between a Weibull median and the desired median times was optimized in order to define the parameters of the Weibull distribution that return these median times for each arm:

$$\text{median}\left( \boldsymbol{T}_{Wei} \right)_{j}=\left( \lambda_{j}\exp\left( E\left[ \boldsymbol{X}^{'}\boldsymbol{\beta} \right] \right) \right)^{-1/\nu_{j}}*{\log\left( 2 \right)}^{1/\nu_{j}}$$

$$\min_{(\lambda_{j},\nu_{j})} \left( median\left( \boldsymbol{T}_{Wei} \right)_{j} - m_{j} \right)^{2}$$

The *β* in the above expression are the coefficients estimated on the training subset of the TROPIC data. They are -0.25, 0.38, 0.65, -0.005, -0.03, 0.2 respectively for covariates ARM, ECOG, PROG, AGE, HGB, and LALP. Only these covariates were used in this estimation for simplicity and to allow for greater variability. Once the Weibull parameters λ, υ were estimated, the simulated event times were drawn as specified for the Cox-Weibull model in Bender et al (2005)^1^:

$$t_{evt,j}=\lambda_{j}\left( -\log UNIF\left( n \right)*\exp\left( -\boldsymbol{X}'\boldsymbol{\beta} \right) \right)^{1/\nu_{j}}.$$

The censoring times were also drawn from a Weibull distribution with shape parameters set equal to those optimized for the event times and with scale parameters chosen by trial and error to result in the desired levels of censoring. The shape parameters used were the following: 10% censoring used shape values of 48, 46.8, 50.6 for sample sizes 200, 500, and 1000 respectively; 30% censoring used shape values 23.85, 24.6, and 24.6 for sample sizes 200, 500, 1000 respectively.

The observed time-to-event variable was defined per usual as the minimum of event time and censoring time: *t_i_* = min{*t_evt,i_*, *t_cens,i_*} with assignment to *t_evt_* when event and censoring time are equal.

**Section 2.** *Missing Data Mechanism*

To achieve missing at random the choice of which observations of the incomplete variables would be missing has to depend on the complete covariates only. A logistic regression model was employed to regress the probability of missing on the complete covariates – all those listed above except HBG, LALP, and PROG - as well as EVENT:

$$\text{logit}(\Pr\left( NA \right))= \text{logit}\left( \pi\right)=\boldsymbol{W}'\boldsymbol{g}$$

where ***W*** = (1, complete categorical covariates, complete centered continuous covariates, EVENT) and *π* is the probability that HGB, LALP or PROG is missing or not available (*NA*). $\boldsymbol{g}$ was found by numerical optimization to attain the desired expected probability of missingness for the given simulation scenario: $M$ = 0.05, 0.10, or 0.15. To be explicit, the square difference between the average logistic regression value and $M$ was minimized over $\boldsymbol{g}$:

$$\min_{\boldsymbol{g}} \left( E\left[ \frac{\text{exp}\left( \boldsymbol{W}'\boldsymbol{g} \right)}{1+exp(\boldsymbol{W}'\boldsymbol{g})} \right]-M \right)^{2}$$

The estimated parameters $\hat{\boldsymbol{g}}$ were then recursively used to estimate a probability of missingness $\hat{p}$ for each observation. These estimated probabilities were subsequently used in Bernoulli draws to set the observation to missing. This draw was repeated for each incomplete covariate with the same $\hat{p}$. To control the percent missingness across the simulation datasets more tightly, when the number of potential *NA*’s exceeded the expected count of missing values (i.e. current *NA* count *>* $M$**N*), a random sample of the expected count size was drawn from the current set of *NA* observations.

For reference, the correlation between missing values and the complete covariates in the TROPIC training set are shown in Table 2 below.

**References**

1. De Bono J, Oudard S, Ozguroglu M, et al. Prednisone plus cabazitaxel or mitoxantrone for metastatic castration-resistant prostate cancer progressing after docetaxel treatment: a randomised open-label trial. *Lancet* 2010; 376: 1147–1154.
2. Bender R, Augustin T and Blettner M. Generating survival times to simulate Cox proportional hazards models. *SIM* 2005; 24: 1713-1723.
3. Halabi S and Singh B. Sample size determination for comparing several survival curves with unequal allocations. *SIM* 2004; 23: 1793-1815.

Table 1. Correlation among covariates in both TROPIC and simulated data

|  | ECOG | PROG | PAIN | BMI | WHITE | CHEMO | MEAS_DIS | AGE | HGB | LALP | LPSA | TIME_ HORMONE | YR SINCE DIAG |
| --- | --- | --- | --- | --- | --- | --- | --- | --- | --- | --- | --- | --- | --- |
| ECOG | 1.00 | 0.03 | 0.25 | -0.04 | -0.04 | -0.09 | 0.02 | 0.12 | -0.23 | 0.15 | 0.02 | -0.03 | -0.01 |
| PROG | 0.03 | 1.00 | 0.08 | 0.07 | -0.11 | 0.09 | -0.02 | -0.04 | -0.16 | 0.12 | 0.21 | -0.07 | -0.06 |
| PAIN | 0.25 | 0.08 | 1.00 | -0.05 | 0.03 | 0.08 | -0.03 | -0.13 | -0.25 | 0.30 | 0.20 | -0.07 | -0.13 |
| BMI | -0.04 | 0.07 | -0.05 | 1.00 | 0.12 | -0.04 | 0.01 | -0.17 | 0.12 | -0.13 | -0.06 | 0.00 | 0.09 |
| WHITE | -0.04 | -0.11 | 0.03 | 0.12 | 1.00 | 0.09 | 0.06 | 0.00 | 0.05 | 0.05 | 0.00 | 0.04 | 0.00 |
| CHEMO | -0.09 | 0.09 | 0.08 | -0.04 | 0.09 | 1.00 | 0.05 | -0.03 | 0.05 | 0.05 | 0.13 | 0.18 | 0.13 |
| MEAS_DIS | 0.02 | -0.02 | -0.03 | 0.01 | 0.06 | 0.05 | 1.00 | -0.09 | -0.09 | -0.12 | -0.02 | 0.11 | 0.08 |
| AGE | 0.12 | -0.04 | -0.13 | -0.17 | 0.00 | -0.03 | -0.09 | 1.00 | -0.13 | -0.04 | -0.01 | 0.18 | 0.32 |
| HGB | -0.23 | -0.16 | -0.25 | 0.12 | 0.05 | 0.05 | -0.09 | -0.13 | 1.00 | -0.22 | -0.29 | 0.08 | 0.04 |
| LALP | 0.15 | 0.12 | 0.30 | -0.13 | 0.05 | 0.05 | -0.12 | -0.04 | -0.22 | 1.00 | 0.36 | -0.15 | -0.16 |
| LPSA | 0.02 | 0.21 | 0.20 | -0.06 | 0.00 | 0.13 | -0.02 | -0.01 | -0.29 | 0.36 | 1.00 | 0.08 | -0.02 |
| TIME_HORMONE | -0.03 | -0.07 | -0.07 | 0.00 | 0.04 | 0.18 | 0.11 | 0.18 | 0.08 | -0.15 | 0.08 | 1.00 | 0.68 |
| YRSINCEDIAG | -0.01 | -0.06 | -0.13 | 0.09 | 0.00 | 0.13 | 0.08 | 0.32 | 0.04 | -0.16 | -0.02 | 0.68 | 1.00 |

Table 2. Correlation between complete covariates and missing values in TROPIC

|  | AGE | MEAS_DIS | ECOG | CHEMO | PROG | WHITE | Bone mets | Visceral mets |
| --- | --- | --- | --- | --- | --- | --- | --- | --- |
| M. B | 0.032 | 0.041 | -0.055 | -0.030 | 0.016 | 0.020 | 0.020 | -0.025 |
| M. PAIN | -0.014 | 0.034 | -0.086 | -0.023 | 0.033 | 0.151 | -0.081 | -0.015 |
| M. HGB | -0.041 | -0.040 | -0.024 | -0.052 | 0.044 | 0.013 | -0.072 | 0.001 |
| M. TIME_HORMONE | 0.078 | 0.086 | 0.008 | -0.033 | -0.041 | -0.006 | -0.006 | 0.046 |
| M. YRSINCEDIAG | -0.009 | -0.102 | -0.085 | -0.006 | 0.029 | 0.008 | -0.012 | -0.045 |
| M. LPSA | -0.026 | -0.024 | -0.016 | -0.060 | 0.047 | 0.019 | -0.062 | -0.008 |
| M. LALP | -0.036 | -0.049 | -0.033 | -0.030 | 0.057 | 0.038 | -0.029 | -0.006 |
| M. X stands for missing values in variable X | | | | | | | | |
